# Long-term evaluation on urban intensive land use in five fast-growing cities of northern China with GEE support

**DOI:** 10.1038/s41598-021-00285-8

**Published:** 2021-10-20

**Authors:** Yiqun Shang, Xinqi Zheng, Rongqing Han, Wenchao Liu, Fei Xiao

**Affiliations:** 1 School of Information Engineering, China University of Geosciences, Beijing, 100083 China; 2 College of Geography and Environment, Shandong Normal University, Jinan, 250358 China; 3 Information Center of Ministry of Natural Resources of the People’s Republic of China, Beijing, 100830 China; 4 Technology Innovation Center for Territory Spatial Big-Data, MNR of China, Beijing, 100830 China

**Keywords:** Environmental economics, Sustainability

## Abstract

Intensive land use (ILU) is a multi-objective optimization process that aims to simultaneously improve the economic, social, and ecological benefits, as well as the carrying capacity of the land, without increasing additional land, and evaluation of the ILU over long time series has a guiding significance for rational land use. To tackle inefficient extraction of information, subjective selection of dominant factor, and lack of prediction in previous evaluation studies, this paper proposes a novel framework for evaluation and analysis of ILU by, first, using Google Earth Engine (GEE) to extract cities’ built-up land information, second, by constructing an index system that links economic, social and ecological aspects to evaluate the ILU degree, third, by applying Geodetector to identify the dominant factor on the ILU, finally, by using the S-curve to predict the degree. Based on the case study data from northern China’s five fast-growing cities (i.e., Beijing, Tianjin, Shijiazhuang, Jinan, Zhengzhou), the findings show that the ILU degree for all cities has increased over the past 30 years, with the highest growth rate between 2000 and 2010. Beijing had the highest degree in 2018, followed by Tianjin, Zhengzhou, Jinan, and Shijiazhuang. In terms of the time dimension, the dominant factor for all cities shifted from the output-value proportion of secondary and tertiary industries in the early stage to the economic density in the late stage. In terms of the space dimension, the dominant factor varied from cities. It is worth noting that economic density was the dominant factor in the two high-level ILU cities, Beijing and Tianjin, indicating that economic strength is the main driver of the ILU. Moreover, cities with high-level ILU at the current stage will grow slowly in the ILU degree from 2020 to 2035, while Zhengzhou and Jinan, whose ILU has been in the midstream recently, will grow the most among the cities.

## Introduction

Since the 1990s, China has experienced massive population growth and rapid socio-economic development, resulting in numerous challenges, particularly land problems^1^. These problems are especially severe in northern China^2^. In this context, the shortage of land resources has gradually become a key factor restricting urban development. In order to optimize the land use structure and improve the land use efficiency, intensive land use (ILU) has become an important strategy to alleviate many conflicts, including the conflict between people and land. It is also an inevitable choice for the development of urbanization in China^3^. Specifically, the ILU refers to meeting the appropriate scale of urban development under the current conditions, taking the rational layout of the city, optimization of land use structure and sustainable development as the premise, and continuously improving the land use efficiency and achieving good economic, social and ecological benefits through increasing the element investment level of the unit land area and improving the land management^4–7^. The definition shows that the ILU is a complex process and assessing it requires knowledge from economics, social sciences, urban planning, geography, ecology, and statistics, demonstrating its interdisciplinary character. Therefore, this complexity and the inherent interdisciplinary characteristics of the ILU determines the difficulty of the comprehensive evaluation. How to scientifically evaluate and analyze the ILU, to provide references for Sustainable Development Goals (SDGs) has become a hot topic in recent years^5,8,9^.

Recently, a considerable amount of literature has grown up around the theme of evaluation for ILU. From the perspective of spatial scales, the evaluation scales in existing studies are various, mainly including national, provincial, municipal, and village regions, as well as specific land use types such as industrial land and educational land^3,5,8–12^. From the perspective of temporal scales, the evaluation scales cover time nodes and time series. Meanwhile, a rational and scientific ILU evaluation index system serves as the foundation for accurately evaluating the ILU degree in a city. The evaluation system often relies on spatial scales and research objects. However, the evaluation involves many indices, including both socio-economic and natural types, so it is also vital to select indices with reliable and easily accessible data sources. The traditional evaluation of the ILU degree frequently relies on statistical information, which has some drawbacks in extracting the index data required for the evaluation^13^. For instance, some index data was missing due to incomplete statistics in the early years, and some data from different years had different statistical scales. Because of the difficulties in extracting reliable index information, evaluating the degree in long time series is challenging.

With the development of satellite remote sensing, carrying out research on ground objects (e.g., built-up land, cropland) has already been possible under the long time series, so making full use of information extracted from satellite images for evaluation can effectively alleviate these problems from statistical data. At present, the application of machine learning (ML) algorithms to land use classification has attracted much attention. ML algorithms can be divided into two main subtypes: unsupervised and supervised algorithms^14^. Unsupervised algorithms include fuzzy C-means algorithm, K-means algorithm and ISODATA^15^. As they do not allow expert knowledge input and their classification results are challenging to match with the actual situation, they are not commonly selected for use. While the main representatives of supervised algorithms are support vector machines (SVM) and maximum likelihood classifiers (MLC)^16^. They also have some drawbacks. For instance, SVM requires complex parameter settings before classification, while MLC can only perform more accurately if the spectral data satisfy a normal distribution. To address these challenges, scholars have proposed more advanced algorithms^15^, such as Random Forest (RF) and Classification and Regression Trees (CART). Among them, RF is currently the most popular algorithm, and this is because it can achieve higher accuracy than the above algorithms and require less parameter tuning^17^. Hence, we selected RF as the classifier in this paper. However, the traditional classification process often relies on the local computer, which requires downloading satellite images to the local computers and configuring the software environment. The operation steps are relatively complicated, and the classification efficiency is primarily dependent on computer performance. The Google Earth Engine (GEE) platform, combines cloud computing power with massive multi-source satellite data and can efficiently investigate scientific topics at various spatial and temporal scales^18–20^. It is valuable to map land use effectively and provides a chance to solve the problems listed above. Hence, using the GEE platform to extract land use information in conjunction with existing reliable socio-economic data is helpful for efficient ILU assessment.

Analysis of the dominant factor affecting the ILU is essential for understanding the inner mechanism and operation law of ILU in cities. Notably, many approaches reveal the driving forces underlying geographical phenomena^21–23^, such as correlation analysis, partial least squares regression, and multivariate regression analysis. However, given the spatial heterogeneity of geographical phenomena and the complex process of the phenomenon response to the influencing factors, the inflexible linear models are unlikely to accurately reveal the inner mechanism between the factor and the phenomenon, and the presence of exogenous covariates (e.g., individual choices, policy regulations) can also limit the usefulness of the model^24^. To address these issues, Geodetector, based on the theory of spatial stratified heterogeneity, was proposed to measure spatial stratified heterogeneity and explore the driving forces of factors on the phenomenon^25^. As a result, Geodetector offers an opportunity to explore the dominant factor of the ILU degree.

In addition, the prediction of the ILU is significant in guiding future urban development. However, current ILU studies have mainly focused on the study area’s past ILU level. Specifically, scholars calculated the study area’s ILU in the past to analyze changes in the same region or differences between regions. Although prediction can better guide the ILU, few studies have focused on predicting the future ILU through the past ILU degree. Therefore, this paper explores the method of predicting ILU degree by drawing on forecasting studies related to cities. Specifically, Bettencourt^26^ developed a theoretical framework to predict cities’ properties as a series of scaling relations applicable to all urban systems. This finding inspires scholars to find suitable functions to predict the cities’ properties in the future. Thus, the key to predicting the ILU level is to find a suitable function. Moreover, it is worth noting that many studies^27,28^ have shown that urbanization evolution in a city can be expressed as an S-curve; this curve shows an initial slow growth followed by rapid growth and finally a slow growth again. As an indirect indicator to assess the quality of the urbanization evolution, the ILU degree can also theoretically be modeled using the S-curve. This finding will provide references for subsequent ILU prediction studies and give better scientific support for land use optimization.

**Figure 1:**
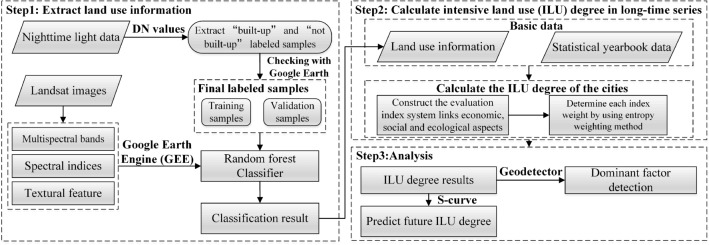
Research framework of this paper.

Based on the above literature review, we can find the problems in current ILU evaluation studies, including difficulties in extracting index information under long time series, subjective analysis of the dominant factor on ILU, and lack of prediction. To address these issues, this paper proposes a research framework (Fig. 1) for evaluation and analysis of the ILU. It mainly includes, first, selecting the “built-up” and “not built-up” labeled samples through nighttime light (NTL) data and using GEE to extract cities’ built-up land information based on the Landsat images; second, constructing an index system that links economic, social, and ecological aspects to evaluate the ILU degree; third, applying Geodetector to identify the dominant factor on the ILU; finally, using the S-curve to predict the degree.

## Materials and methods

### Study area

**Figure 2:**
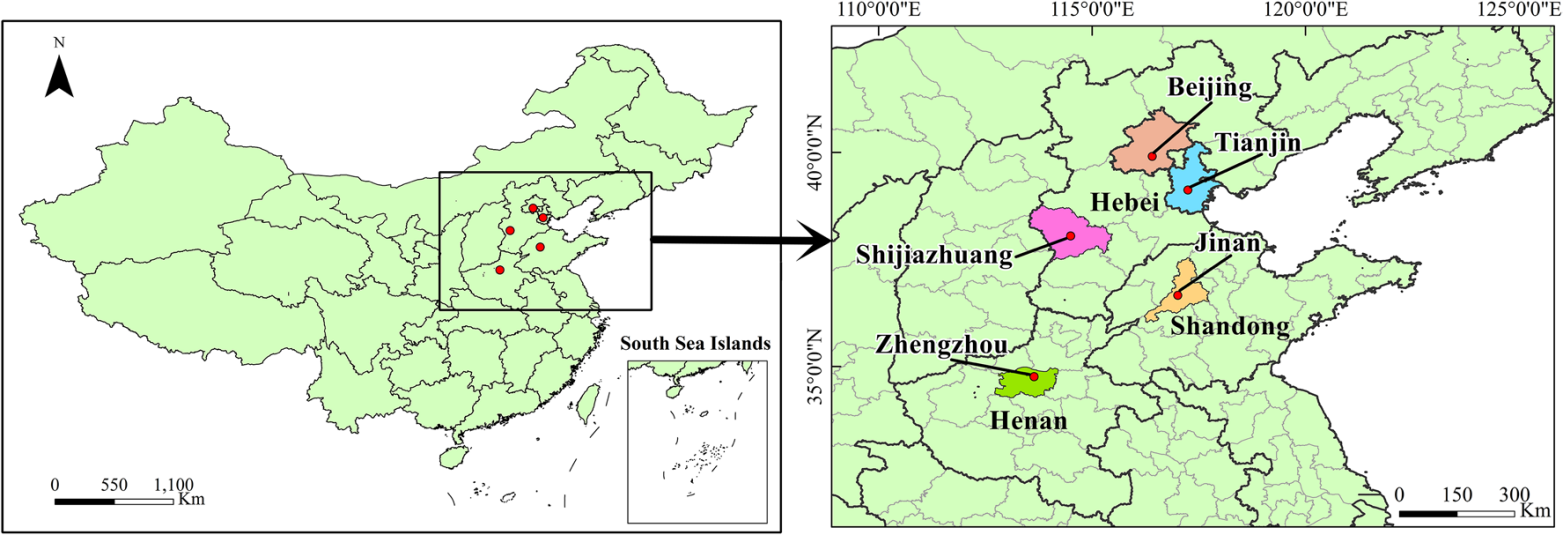
Spatial location of study area. Map created using ArcGIS.

Northern China’s fast economic development and urbanization have spurred population expansion and exacerbated the tension between land supply and demand over the past 30 years. In order to encourage high-quality urban development, it is important to choose representative cities for ILU evaluation and analysis. Hence, we considered the northern cities' economic, demographic, and geographic conditions, and selected five cities with high overall strength, including Beijing, Tianjin, Shijiazhuang, Jinan, and Zhengzhou (Fig. 2). They play an essential role in guiding regional development and carrying out national strategic goals. Specifically, Beijing and Tianjin are the two municipal cities, which are politically equal to provinces. Shijiazhuang, Jinan, and Zhengzhou are the provincial capitals of Hebei, Shandong, and Henan provinces, respectively. As the center of politics, culture, and high technology, Beijing covers 16,410 km^2^, with a population of 21.54 million and a GDP per capita of 23,135 USD in 2018. Tianjin is the largest port city and economic center in northern China, and it covers 11,903 km^2^, with a population of 15.59 million and a GDP per capita of 18,242 USD in 2018. Shijiazhuang is the political, economic, scientific center of Hebei and a commercial port in northern China. It has an area of 14,530 km^2^, with a population of 10.95 million and a GDP per capita of 8421 USD in 2018. Jinan is located in the middle of Shandong, a famous national historical and cultural city in China. It covers 7998 km^2^, with a population of 6.55 million and a GDP per capita of 16,064 USD in 2018. Zhengzhou is the core city of the Central Plains City Cluster, an important national integrated transportation hub. It covers 7509 km^2^, with a population of 10.13 million and a GDP per capita of 16,111 USD in 2018.

### Landsat image data

In order to extract the information of built-up land in the study area, we utilized the time series of Landsat Tier 1 Top-of-Atmosphere (TOA) reflectance data acquired for 1990, 1995, 2000, 2005, 2010, 2015, and 2018 via GEE (https://earthengine.google.org). Due to the long time series involved in this study, TOA data from the Landsat 5 satellite and the Landsat 8 satellite were employed, both of which contain common bands of Blue, Green, Red, NIR, and two short-wave infrared bands (SWIR_1_ and SWIR_2_) with a spatial resolution of 30 m. We selected the image data from June to September as the original image set. In order to make the most of all acquired images and create the annual cloud-free Landsat composites at each location, we applied the algorithm “simpleCloudScore” for the TOA data, and the least cloudy pixels were taken as the composite value. The algorithm can evaluate the “cloud score” by integrating indices such as the Normalized Difference Snow Index (NDSI), the brightness and temperature from the Landsat TOA reflectance data^29,30^. Finally, we successfully generated the least cloud images covering the study area from 1990 to 2018.

### Nighttime light (NTL) data and labeled samples selection

In this study, the land use information required for the ILU evaluation is mainly the built-up land information, so we only mapped the “built-up” land and “not built-up” land during the classification process. The NTL data were used to select “built-up” and “not built-up” labeled examples^31^. Given the long time series of this study, a single NTL product cannot cover the entire study period, so we used two sources of NTL product, covering two different periods. For the period 1992–2013, we used the DMSP/OLS Nighttime Lights Time Series dataset (version 4), which has a unique capability to detect visible and near-infrared emission sources at night with a spatial resolution of 30 arc seconds. The “stable lights” band was used, as it has been processed to discard transient events such as fires, so the background noise is discarded and replaced with zero values. Since the DMSP/OLS data is not available for 1990, we used the closest data to 1992 as a proxy. For the period 2014–2018, we used the VIIRS Day/Night Band Nighttime Lights dataset (version 1), which is composited monthly at a resolution of 15 arc seconds and uses a procedure to correct for stray light. The “avg_rad” band that represents average Day/Night Band radiance values was used. Meanwhile, to maintain consistency in spatial resolution between the two products (i.e., DMSP/OLS data and VIIRS data), we projected them to Lambert Conformal Conic projection and resampled them to 500 m.

**Figure 3:**
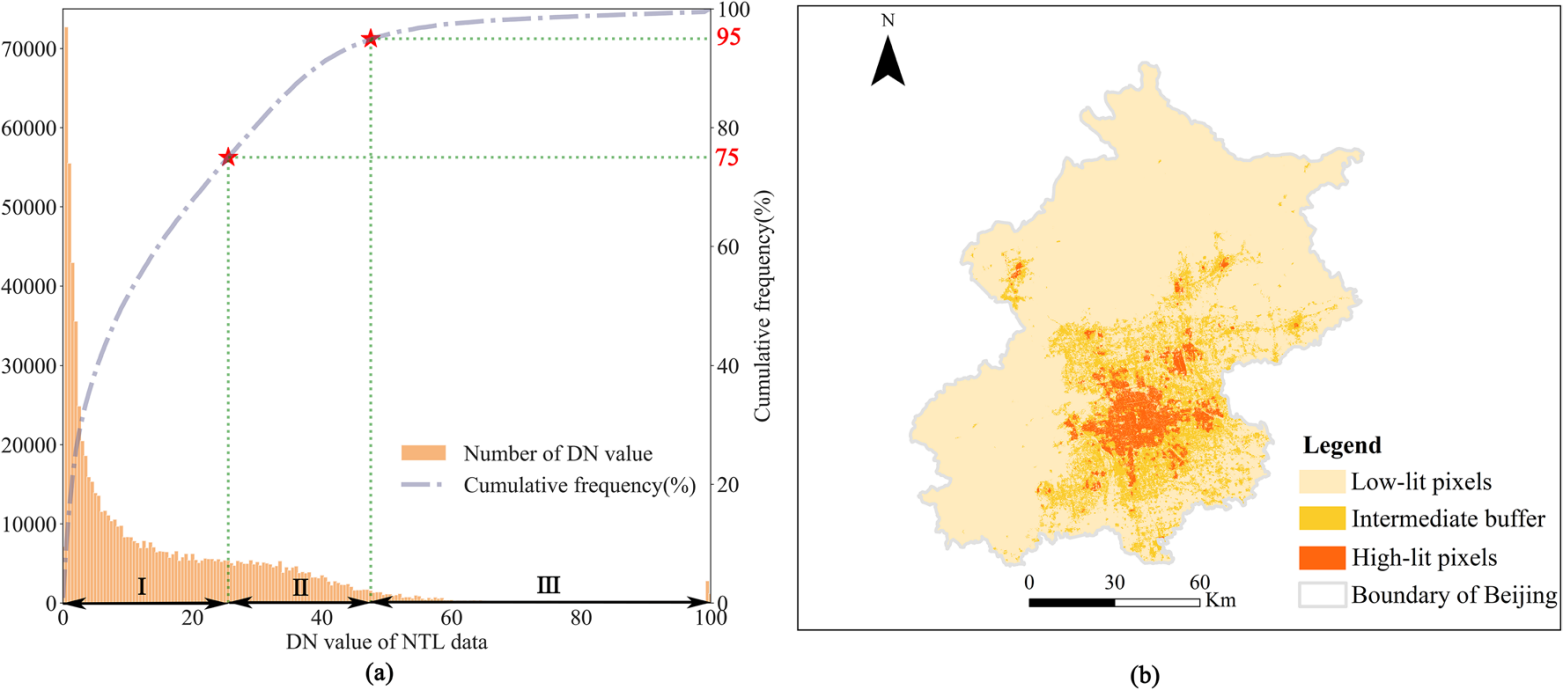
The histogram statistics and spatial result of Beijing’s NTL data, which was obtained from VIIRS Nighttime Lights dataset in 2018. (**a**) The histogram statistics of DN values and its cumulative frequency; (**b**) spatial result of DN values divided into three parts after the operations.

Since the study area contains five cities, manual selection of sample sets would be a time-consuming task. Instead, we applied a new sample selection strategy based on the NTL data, which requires minimal manual input^31^. As pixels with high brightness are associated with artificial structures that emit light, we assume that pixels with DN values above the threshold can represent areas with built-up land. Here, we illustrate the operation steps of Beijing’s NTL data in 2018 as an example, with the following: first, the distribution of DN values was created by using a histogram in GEE, then the cumulative frequency was calculated based on the histogram. By referring to the empirical thresholds^31^, we divided the cumulative frequency into three parts: 0–75%, 75–95%, and 95–100% (Fig. 3a), and the three parts correspond to the three ranges of DN values, such as I represents the range of low-lit pixels, II represents that of the intermediate buffer, and III represents that of high-lit pixels. Limited by the over-saturation effect and blooming effect of NTL data, pixels identified as high-lit may potentially include “not built-up” land cover (e.g., vegetation and water bodies). As a result, we removed these land cover types from high-lit pixels based on Landsat images’ per-pixel NDVI and MNDWI values^31^. Specifically, by using the Otsu method for NDVI (MNDWI)^32^, we got its threshold for identifying vegetation (water bodies). If a pixel’s NDVI (MNDWI) value is higher than the threshold, we identified it as vegetation (water bodies). Then the two types were removed according to the thresholds. Figure 3b shows the spatial result after the above operations. Hence we generated the initial samples of “built-up” land and “not built-up” land by using tools of ArcGIS software, where “built-up” land samples were produced in the spatial range of high-lit pixels, and “not built-up” land samples were acquired in the low-lit pixels. Due to the inevitable errors, these initial samples should also be checked against Google Earth to delete inaccurate samples and add samples where appropriate. We processed the NTL data of each city in different years according to the above steps and finally generated the corresponding labeled samples.

For the subsequent classification of built-up land from 1990 to 2018 for each city, 1200 samples were generated for each year, with a total of 8400 for each city over the seven years (1990, 1995, 2000, 2005, 2010, 2015, and 2018). 70% of the samples were used for training, and the remaining 30% were used for validation.

### Index selection and socio-economic data

Given that land is the spatial carrier of urban economic, social, and ecological systems, and also combined with the definition of ILU, it is clear that ILU is not only about high-intensity input and high-efficiency output, but also the unification of economic, social and ecological benefits, ultimately achieving the coordinated development of economy, society and ecology. Therefore, this paper constructed the evaluation index system for the ILU degree based on three aspects: economic, social and ecological. We adequately selected representative and accessible indices by referring to related studies^4–9^ and the conditions of the study area. Table 1 shows the eight indices that were chosen for the evaluation. The data for these indices are mainly from the “China City Statistical Yearbook” and the statistical yearbooks of the five cities from 1991 to 2019. In addition, some data were retrieved from the China Economic and Social Big Data Research Platform or the official websites of relevant statistical bureaus. All the data format and precision were unified and standardized.

**Table 1:** Evaluation index system of ILU degree.

Type of indices	Index	Character	Calculation method
Economic	Economic density*	+	Output-value of secondary and tertiary industries/built-up land area
	Output-value proportion of secondary and tertiary industries	+	Output-value of secondary and tertiary industries/GDP
	Employee density*	+	Number of employee in secondary and tertiary industries/built-up land area
Social	Population density*	+	Number of resident population/built-up land area
	Proportion of built-up land*	−	Built-up land area/city’s area
	Road network density	+	Total length of urban roads/city’s area
Ecological	Public green space per capita	+	Green space area/number of resident population
	Average industrial wastewater discharge on land*	−	Total industrial wastewater discharge/built-up land area

The index marked with * were calculated by extracting built-up land information in the GEE platform, while all other indices were taken from the statistical data. The “+” in the Character column means a positive index, and “−” means a negative index.

### Random forest (RF) classifier

Since the RF algorithm consists of multiple decision tree classifiers, it outperforms other popular algorithms in many application scenarios (e.g., SVM or kNN). Up to now, RF is one of the most widely used algorithms for land cover classification by using satellite image data^29,30,33^. Furthermore, RF is a machine learning algorithm built into the GEE platform. As a result, we chose RF to extract the built-up land. Based on the experiences of previous studies^34,35^ and data collected, we set ntree to 500, and mtry was set to the default value (sqrt of the total number of all input features). Next, we selected nine features as input features, specifically, five spectral features, i.e., gray values of Blue, Green, Red, NIR, and SWIR1 bands were selected, one textural feature, namely Geary’s C coefficient^36^ was chosen, three spectral indices, including NDVI^37^, MNDWI^38^, and NDBI^39^ were singled out. Table 2 shows the specific information.

**Table 2:** The description of the selected features.

Feature	Formula	Description	References
Geary’s C coefficient	$\frac{(n-1){\sum }_{i=1}^{n}{\sum }_{j=1}^{n}{w}_{ij}{({x}_{i}-{x}_{j})}^{2}}{2{\sum }_{i=1}^{n}{\sum }_{j=1}^{n}{w}_{ij}{\sum }_{i=1}^{n}{({x}_{i}-\overline{x })}^{2}}$	Where *w*_*ij*_ is the spatial weight matrix between units *i* and *j*, *d* is the distance, and *x*_*i*_ and *x*_*j*_ are the attributes of units *i* and *j*, respectively. Here, texture feature was extracted by selecting the NIR band	^36^
NDVI	$\frac{NIR-RED}{NIR+RED}$	Where *NIR*, *RED*, *GREEN* and *SWIR*_*1*_ are the reflectance values for the near infrared, red, green and shortwave infrared bands of the images, respectively	^40^
MNDWI	$\frac{GREEN-SWI{R}_{1}}{GREEN+SWI{R}_{1}}$		^38^
NDBI	$\frac{SWI{R}_{1}-NIR}{SWI{R}_{1}+NIR}$		^41^

The validation process was carried out based on the selected samples using the NTL data and Google Earth. Several accuracy assessment metrics were calculated to assess the accuracy of the extraction. Traditional accuracy assessment often uses overall accuracy (OA) and the Kappa coefficient to indicate whether the classification results are good or bad. However, a growing number of scholars have accepted that the Kappa coefficient has significant limitations and is somewhat misleading, so we also calculate the quantity and allocation disagreement^42^ as alternatives to Kappa. Specifically, the quantity disagreement describes how well the mapped products represent the area covered by each class, and allocation disagreement refers to the spatial agreement between mapped areas versus the validation data. More details of these metrics can be found in the published studies^43,44^. The equations of OA and Kappa are as follows:

1 $$\mathrm{OA}=\frac{{\sum }_{i=1}^{k}{x}_{ii}}{x}$$

2 $$\mathrm{Kappa}=\frac{x{\sum }_{i=1}^{k}{x}_{ii}-{\sum }_{i=1}^{k}{x}_{i*}{x}_{*i}}{x}$$

where *x*_*ii*_ is the number in row *i* and column *i* of the matrix, *x* is the total number of validation samples, and *x*_i*_ and x_*i_ are the total number of samples in row *i* and column *i*, respectively.

### Entropy weighting method

As many methods of determining index weight are susceptible to subjective factors, the entropy weighting method determines weights based on the dispersion of index values, which is relatively objective. This paper refers to the steps of the previous studies^45^ to calculate the corresponding indices’ weights. The steps are as follows:

1. The index data should be subjected to dimensionless processing to normalize the extreme differences, and this step can eliminate the influence of the dimension. The *j-*th index for the i-th city in the λ-th year can be expressed in terms of *x*_*λij*_ (1 ≤ λ ≤ h, 1 ≤ i ≤ m, 1 ≤ j ≤ n, in this paper, h, m and n are 7, 5 and 8 respectively). Equation (3) is used to normalize the positive indices, and Eq. (4) is for the negative indices. Equation (5) is used to normalize all the *X*_*λij*_
  3 $${X}_{\lambda ij}=\frac{{x}_{\lambda ij}-{x}_{min}}{{x}_{max}-{x}_{min}}$$
  4 $${X}_{\lambda ij}=\frac{{x}_{max}-{x}_{\lambda ij}}{{x}_{max}-{x}_{min}}$$
  5 $${P}_{\lambda ij}=\frac{{X}_{\lambda ij}}{{\sum }_{\lambda =1}^{\mathrm{h}}\sum_{i=1}^{m}{X}_{\lambda ij}}$$
  where *X*_*λij*_ denotes the value of *x*_*λij*_ after the standard normalization of the extreme deviation, *P*_*λij*_ denotes the normalized value of *X*_*λij*_.
2. Calculate the entropy value of each index, where *k* = 1/ln(*h*·*m*).
  6 $$ E_{j}  =  - k\sum\limits_{{\lambda  = 1}}^{h} {\sum\limits_{{i = 1}}^{m} {P_{{\lambda ij}} \ln P_{{\lambda ij}} } }  $$
3. Calculate the redundancy of the entropy values for each index and the corresponding weights.
  7 $${D}_{j}=1-{E}_{j}$$
  8 $${W}_{j}=\frac{{D}_{j}}{\sum_{j= \text{1} }^{n}{D}_{j}}$$
4. Calculate the ILU degree for each city in each year.
  9 $${S}_{\lambda i}=\sum_{j=1}^{n}{P}_{\lambda ij}\cdot {W}_{j}$$

### Factor detection by geodetector

Geodetector is a statistical tool developed by Wang, which is a set of methods used to reveal stratified heterogeneity and detect the driving factors behind it^25^. The method is based on the assumption that if the independent variable has a significant influence on the dependent variable, the independent and dependent variables’ spatial distribution should be similar. More specifically, according to spatial distributions of these data, the method not only can quantify the relationship between two variables, Y (geographical phenomenon) and X (influencing factor), but it can also explore the interaction relationship between two influencing factors (X_1_ and X_2_) to the geographical phenomenon Y, without assuming linearity of the association. In this paper, we mainly used the function module Factor Detector of Geodetector to reveal the influencing mechanism of the study, which measures the variable X’s explanatory power to the variable Y through q value, shown in Eq. (10)

10 $$q=1-\frac{1}{{\sigma }^{2}H}\sum_{i=1}^{m}{n}_{D,i}{\sigma }^{2}{H}_{D,i}$$

where *q* is the explanatory power of factor *D* on ILU degree *H*, *σ*^*2*^ is the variance of the overall study object’s degree, *n* is the number of cities, *m* is the number of sub-areas, *σ*^*2*^*H*_*D,i*_ is the variance of the sub-area’s degree, *q* is at [0, 1], the larger the *q* value, the greater the explanatory power of this factor, the factor with the highest *q* of all the factors we regard as the dominant factor.

### Prediction of ILU degree by S-curve

Through statistical analysis of ILU degree in each city, we found the degree of cities largely follows a trend of initial slow growth in the early stage, followed by rapid growth, and finally slow growth. Meanwhile, the maximum degree will not exceed 1. Considering the above factors, we found that the degree is broadly in line with the S-curve trend. As a result, we used S-curve to predict the degree in the future^46^. The study periods include 1990, 1995, 2000, 2005, 2010, 2015, and 2018, all years except 2018 are in line with the 5-year incremental trend, and because 2018 is closer to 2020, we used the data from 2018 for 2020. We forecasted each city’s ILU degree for the three time nodes: 2025, 2030, and 2035 using these data. The forecasts were then used to analyze the land-intensive potential of each city. Each city’s calculation steps are as follows.

1. Solve for the ILU degree after logarithmic conversion, shown in Eq. (11)
  11 $$ L_{j}  = {\text{ln}}\left( {\frac{{S_{j} }}{{1 - S_{j} }}} \right) $$
  where *S*_*j*_ is the ILU degree of the city in year *j*.
2. Construct a linear equation between *L*_*j*_ and the year, then use ordinary least squares (OLS) to solve the parameters *a*, *b* (Eq. 12). Then, substitute *a*, *b* into Eq. (13) to calculate *T*_*y*_ for each year. Finally, the predictive value can be solved by the Eq. (14)
  12 $${L}_{j}={a}_{j}+\mathrm{b}$$
  13 $${T}_{\text{y}}=\mathrm{a}{\text{y}}+\mathrm{b}$$
  14 $${Predict}_{\text{y}}=\frac{{\mathrm{e}}^{{T}_{\text{y}}}}{1+{\mathrm{e}}^{{T}_{\text{y}}}}$$
  where *j* = 1990, 1995, …, 2020, and *y* = 1990, 1995, …, 2035.

## Results

### Extraction results of built-up area

**Figure 4:**
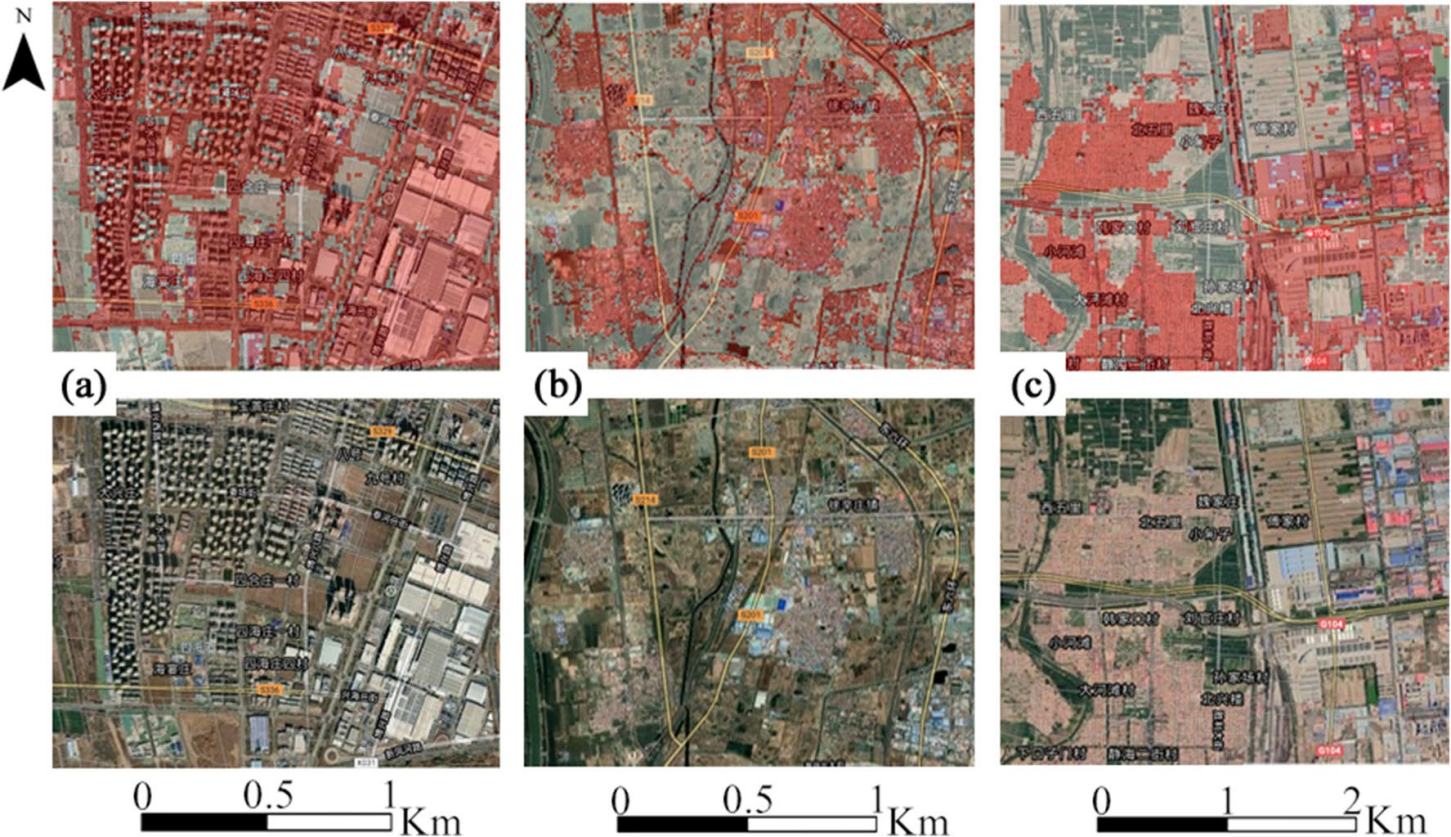
Comparison of local areas before and after classification of satellite images, which were obtained from Landsat-8 OLI images during June to September in 2018. The translucent red part of the first row of the figure shows the extracted built-up land. Map data: Google, ©2021 CNES/Airbus, Landsat/Copernicus, Maxar Technologies.

As the classification results of the built-up land and non-built-up land were identified based on the GEE platform, we selected some local areas to compare and test the classification results (Fig. 4). The translucent red parts of Fig. 4 in the first row show the extracted built-up land sites, including tall buildings, factories, rural settlements, and roads, all of which can be extracted accurately.

**Figure 5:**
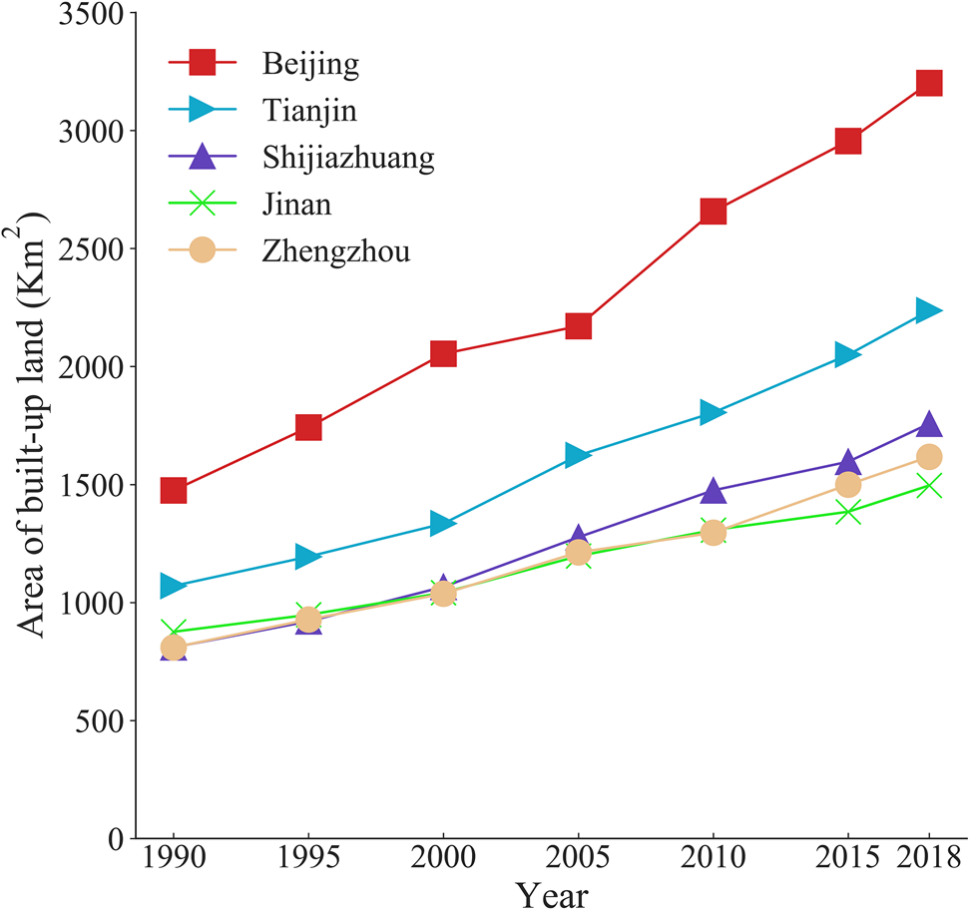
Area of built-up land in each city during 1990–2018.

The accuracy results of the cities during 1990–2018 can be obtained using the validation samples, with the OA of all classification results being above 85%, the Kappa coefficient ranging from 0.73 to 0.85, the allocation disagreement ranging from 7 to 13%, and the quantity disagreement ranging from 2 to 7%. These accuracy results show that the classification results can completely meet the requirements of this study. The area of built-up land for each city from 1990 to 2018 is depicted in Fig. 5, which shows that the built-up land area has steadily increased over the last 30 years, with the volume of built-up land growth varying between cities, with Beijing having the largest increase in built-up land area of 1720 km^2^, followed by Tianjin, Shijiazhuang, Zhengzhou, and Jinan having the smallest increase in built-up land area of 620 km^2^.

### Evaluation results of the ILU degree

**Figure 6:**
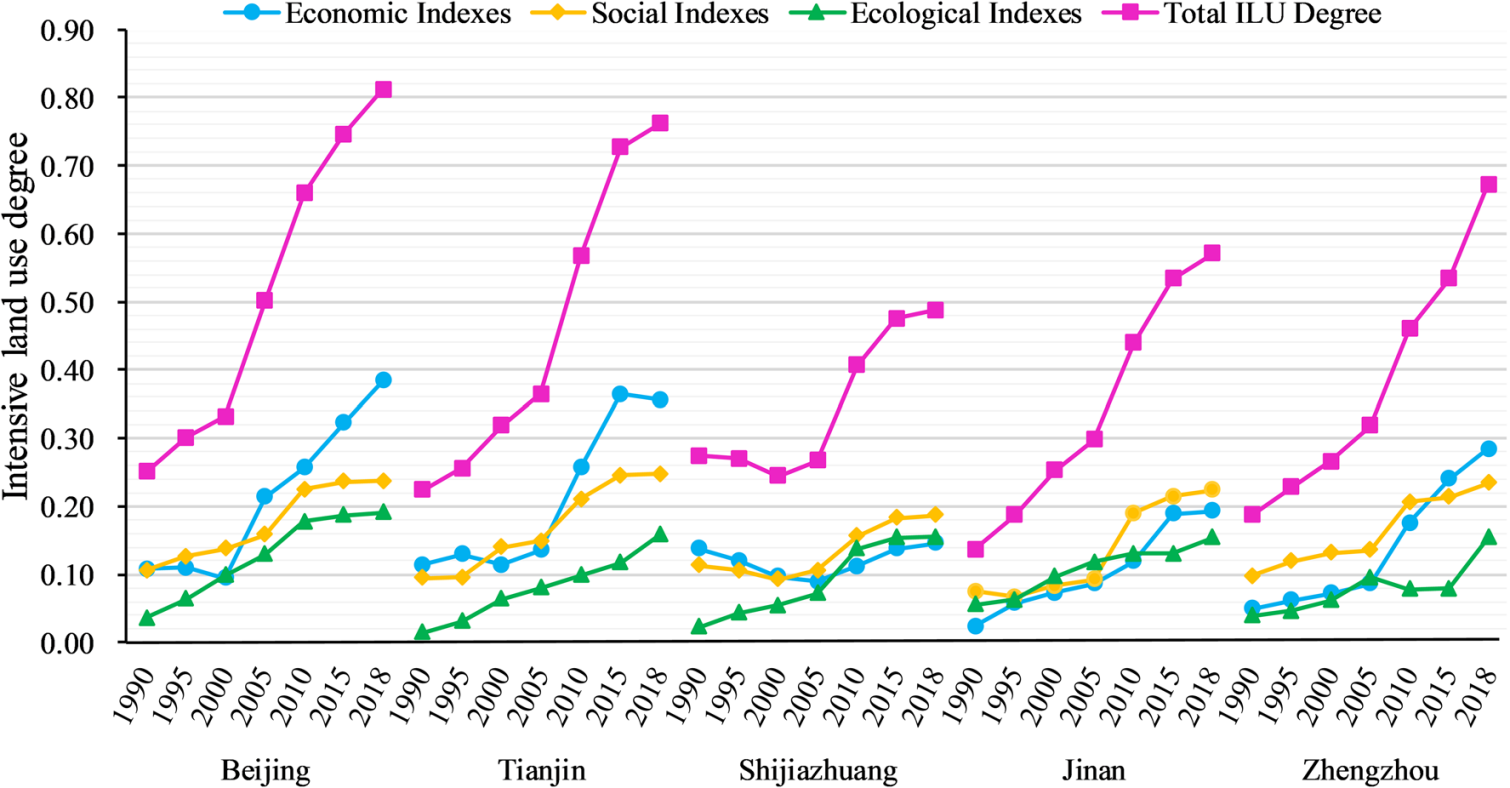
Evaluation result of the ILU degree in each city.

Figure 6 depicts the evaluation result of ILU in each city, with all cities showing an upward trend in ILU between 1990 and 2018. While Shijiazhuang fluctuated, Beijing, Tianjin, Jinan, and Zhengzhou all showed an ascendant trend over time. In 2018, Beijing had the highest level of ILU (0.811), followed by Tianjin (0.762), Zhengzhou (0.672), Jinan (0.571), and Shijiazhuang (0.489). When combined with the cities’ built-up land area, it shows that, despite significant increases in built-up land in Beijing and Tianjin over the last 30 years, their ILU degrees remain high, indicating that both cities have a high level of land resource allocation and efficient land use, which compensates for the high development intensity. Meanwhile, Shijiazhuang also has a more significant increase than Zhengzhou and Jinan in built-up land. However, its current ILU degree is lower than the two cities, indicating that it may be making inefficient use of available built-up land. In terms of the economic, social, and ecological aspects, the degrees for each city in the three aspects have primarily tended to increase over time. However, the increase varies between cities in different aspects. For instance, Beijing, Tianjin, and Zhengzhou have the largest increases, primarily in the economic aspect, whereas Shijiazhuang's economic or social growth degree is much lower than its ecological growth degree. Jinan, in particular, differs from other cities in that it has experienced greater economic and social growth.

**Figure 7:**
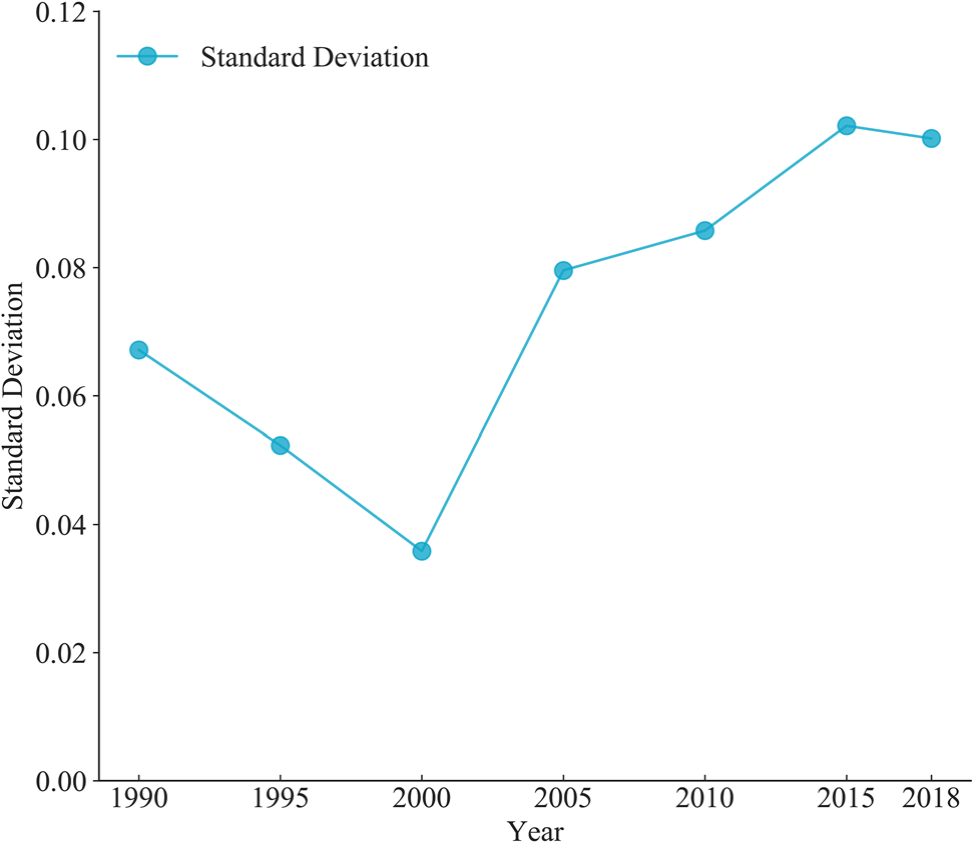
Overall degree of difference between cities from 1990 to 2018.

Table 3 shows the ILU degree growth rate for each city, and it is clear that the rate for each city reached its maximum ratio between 2000 and 2010. Except for Shijiazhuang, which experienced a temporary decline between 1990 and 2000, all other cities have broadly followed the trend of an initial slow growth followed by rapid growth and finally a slow growth again, which is consistent with the S-curve. By quantifying the degree of variation between cities in terms of standard deviation, the result is shown in Fig. 7. The standard deviation across cities decreased between 1990 and 2000, then gradually increased after 2000. Overall, the gap between cities in terms of ILU level has grown over time, with the standard deviation in 2018 being roughly 1.5 times that of 1990, reflecting the growing spatial imbalance in the ILU.

**Table 3:** Growth rate of ILU degree, with the trend in line with the S-curve.

City	1990–2000 (%)	2000–2010 (%)	2010–2018 (%)
Beijing	32.19	98.71	23.14
Tianjin	41.74	78.34	34.33
Shijiazhuang	− 10.81	65.92	20.10
Jinan	63.25	73.29	29.85
Zhengzhou	42.44	72.75	46.26

### Results of geodector

In order to explore the dominant factor affecting the city’s ILU degree in depth, we used the index system’s indices as influencing factors, investigated the dominant factor in time and space dimensions by using Geodetector. The factor with the largest q in each detection was considered as the dominant factor.

1. Exploration of the time dimension.
  We took the five cities as a whole, then utilized Geodetector to explore the dominant factor from two time periods (1990–2000 and 2005–2018). The proportion of secondary and tertiary industries output-value (q = 0.86) was the dominant factor impacting the ILU between 1990 and 2000, suggesting that the rise in the ILU of cities was mostly impacted by the upgrading of industrial structure. After 2000, however, economic density (q = 0.97) became the dominant factor, indicating that the growth during this time was attributable to the strengthening of the economic level of cities and the economic output efficiency of the land.
2. Exploration of the spatial dimension.
  The dominant factor may vary not just through time but also from city to city. Table 4 shows the results of exploring each city during the study period. The dominant factor affecting the ILU in Beijing and Tianjin was economic density, showing that the improvement was primarily dependent on the increase of the economic output efficiency of the land. While development intensity was the dominant factor in Jinan and Zhengzhou, showing the improvement in recent years relied on regulating development intensity and making full use of the existing stock of built-up land to avoid the disorderly expansion. Specifically, the dominant factor for Shijiazhuang was population density, indicating that increasing the ILU relied on maintaining a certain rate of land area expansion. It may achieve high population density and improve the level of ILU by maintaining a high rate of population growth and attracting people to adjacent cities.

**Table 4:** Detection result of the dominant factor for each city.

City	Dominant factor
Beijing	Economic density (q = 0.99)
Tianjin	Economic density (q = 0.96)
Shijiazhuang	Population density (q = 0.99)
Jinan	Development intensity (q = 0.98)
Zhengzhou	Development intensity (q = 0.97)

### Predictive results of ILU degree

**Figure 8:**
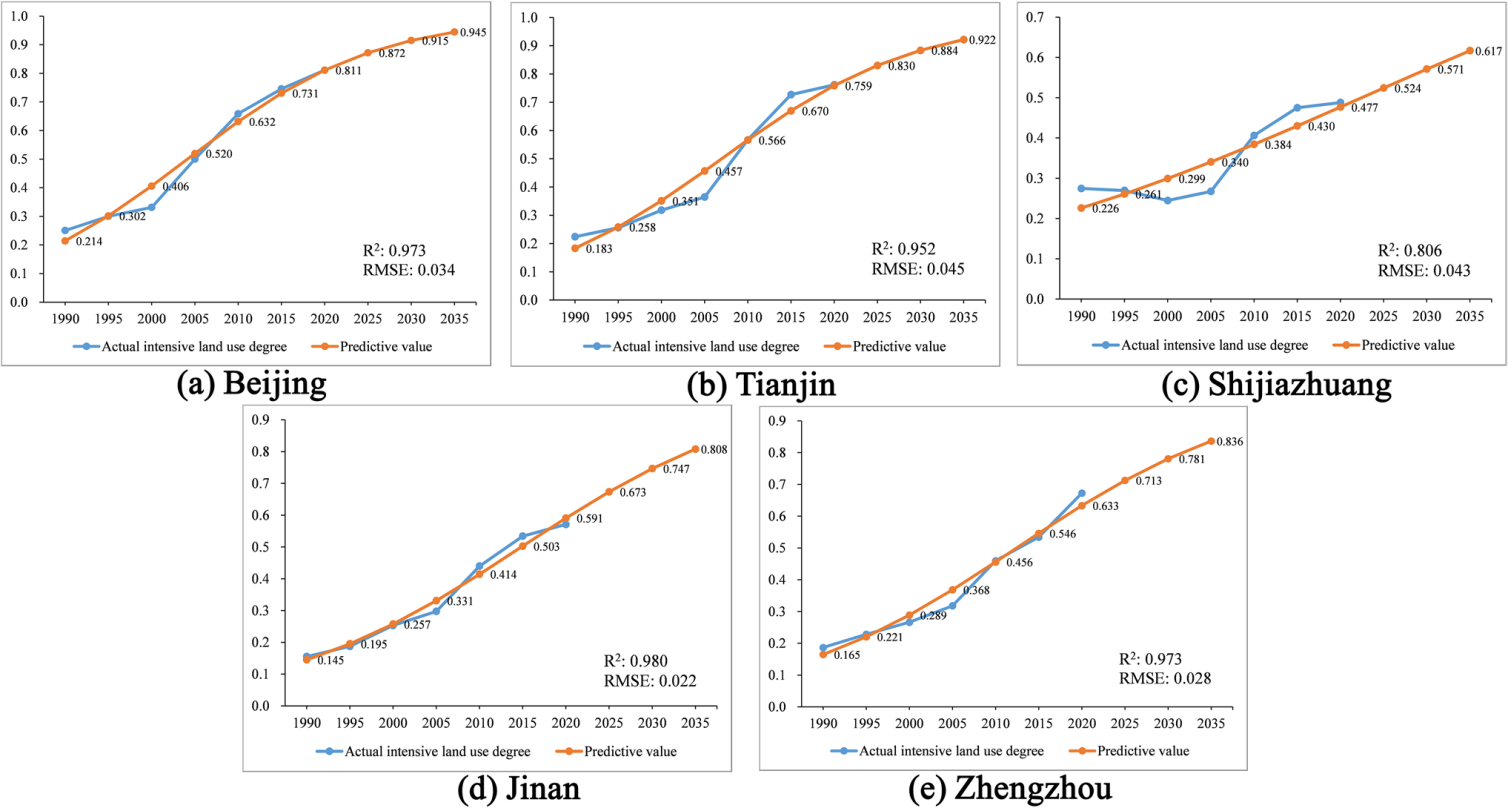
Predictive result of the ILU: (**a**) Beijing; (**b**) Tianjin; (**c**) Shijiazhuang; (**d**) Jinan; (**e**) Zhengzhou.

Using the S-curve to predict the ILU degree, the results of the five cities are shown in Fig. 8. The predictive results from 1990 to 2020 show a good fit by observing the parameters R^2^ and RMSE. R^2^ of all cities is greater than 0.95 except for Shijiazhuang, below 0.9, and RMSE of all cities is lower than 0.05. These evaluation parameters indicate that the forecast results are indicative on the whole.

In terms of time point, Beijing will still have the highest degree of ILU at 0.945 by 2035, followed by Tianjin (0.922), Zhengzhou (0.836), Jinan (0.808), and Shijiazhuang (0.617). This ranking is in the same order as in 2018. The results for Beijing and Tianjin tend the S-curve from 1990 to 2035. While Shijiazhuang, Jinan, and Zhengzhou show a trend close to a line over the same period. By calculating the growth degree between 2020 and 2035, Jinan has the most growth with 0.217, followed by Zhengzhou (0.203), Tianjin (0.163), Shijiazhuang (0.140), and Beijing (0.133). Hence this ranking indicates that Jinan and Zhengzhou are likely to have significant increases in the ILU. In contrast, Beijing's growth will slow down in the future, and Tianjin is essentially similar to Beijing. Furthermore, Shijiazhuang's ILU degree in the prediction is low since it experienced fluctuations during 1990–2005, causing its growth stage to diverge from the S-curve trend.

## Discussion

### Analysis of evaluation results of the ILU

The ILU development trend in the five cities is similar, but there are differences in the ILU degree. The direct reason for the differences is that different cities have different economic, social, or ecological strengths. However, the deep reason is that the dominant factor for ILU development differs between cities. Specifically, the five cities all show a general upward trend in ILU level over the last 30 years, and the growth rate of ILU in each city is greatest between 2000 and 2010. On the other hand, there are differences in the ILU between the cities. Beijing has the highest level of ILU at the current stage, followed by Tianjin, Zhengzhou, Jinan, and Shijiazhuang. In detail, Beijing, Tianjin, and Zhengzhou have the largest increases, mainly in the economic aspect, while Shijiazhuang’s economic or social growth degree is far below its ecological growth degree. Jinan is a different story. Its growth is strong in both economic and social aspects. Thus it is clear that cities making economic progress are more likely to achieve higher ILU degrees. This is due to the fact that ILU is an optimization progress without increasing additional land. This process requires measures such as increasing building density, technology, and management capabilities, and these measures all need a significant capital investment^47^. Hence, increasing the economic strength can ensure these measures are implemented and, as a result, raise the ILU level. The Geodetector results can also validate this hypothesis. Therefore, if cities with a low level of ILU want to enhance their ILU, they should learn from high-level ILU cities. For instance, cities such as Jinan, Zhengzhou cannot rely solely on control of the built-up land scale to raise the ILU level in the future, but also learn from the development experience of Beijing or Tianjin, such as promoting industrial transformation or improving technology and capital investment^48^, thus improving their economic strength and enhancing the development potential.

### Analysis of the predictive results of the ILU

Cities with a high level of ILU will slow down in the future, whereas cities with medium to low levels of ILU will see a significant increase in ILU. Concretely, we can see that Jinan and Zhengzhou will likely achieve a significant increase in ILU degrees during 2020–2035, while Beijing and Tianjin's growth will slow down. Beijing has reached a high level of ILU at the current stage, and Tianjin is broadly similar to Beijing. Shijiazhuang's ILU degree in the forecast is not high because it has experienced fluctuations during 1990–2005, which may prevent S-curve from predicting better. Combining results of time nodes with the growth of periods, we can anticipate that, though there will still be a difference in ILU level between the cities in the future, it should be lower than it is now because results suggest that cities with high ILU at this stage will enter a slow growth phase. In contrast, cities with relatively low-level ILU, which currently have more development potential, will enter a period of rapid growth. Thus the gap between cities will gradually narrow.

### Discussion of the uncertainties

The uncertainties that exist in this study will have an impact on the study’s accuracy. First, the classification results of the Landsat images cannot be completely accurate, introducing uncertainty into the following ILU evaluation. Specifically, there are different types of uncertainties in the various operations from data acquisition, pre-processing, and classification in the classification process. Therefore, this paper attempts to minimize this uncertainty by standardizing the entire classification process as much as possible and ensuring the classification accuracy for each city is above 85%. Second, though the S-curve can predict the future ILU based on the past ILU degree of cities, it cannot avoid the influence of exogenous variables (e.g., future policies of cities), and this uncertainty may have an impact on the model’s prediction. In order to mitigate the impact of this uncertainty, this paper avoids predicting the results too far in the future. Furthermore, this paper is based on the ILU's time-series data trend to forecast so that the forecast results can be in line with reality.

### Research contributions

The main contributions of this study are as follows: (1) It utilizes the GEE platform into extracting information of key indices for evaluating the ILU degree, solving the problem of inefficient and inaccurate extraction of information that has long plagued the evaluation process; (2) it constructs a relatively comprehensive evaluation system by taking three aspects into account: economic, social and ecological, and after calculating the ILU level, it uses Geodetector to detect the dominant factor affecting the ILU, thereby overcoming the shortcomings of subjective selection of the dominant factor in the evaluation; (3) it innovatively uses the S-curve to predict the ILU degree in the future, enriching the theoretical methods to anticipate the ILU.

### Limitations and future work

There are also a few limitations that require further research. One of them is that the number of cities selected in this study is relatively small, which may limit our findings on the spatial characteristics of ILU. Another shortcoming is the limited number of indices selected for assessment in this study due to the limitations of data collection, and the selection can affect the evaluation results of ILU. Hence, in the future, to explore the characteristics of urban ILU at a larger spatial scale and to reveal more factors affecting the level of ILU, we will consider selecting more cities as the study area and including more indices reflecting the ILU. Furthermore, we intend to investigate the fusion and correction of the NTL data to extract the “built-up” land samples with greater accuracy.

## Conclusion and implications

This paper developed an index system based on the economic, social, ecological aspects, rapidly extracted built-up land information with GEE support, and used the entropy weighting method to evaluate the ILU degree by combining socio-economic statistics. Then, we used Geodetector to explore the dominant factor and S-curve to make predictions on the ILU degree, completing a whole framework for the ILU research that included the extraction of information, calculation of the ILU degree, detection of the dominant factor, and prediction. The findings are as follows.

1. Based on the satellite data and the powerful cloud computing capability through the GEE platform, land use classification can be extracted efficiently. The increase in the area of built-up land is an inevitable trend in urban development, but the results of the built-up land and ILU degree in each city show that the built-up land area has an impact on the ILU, but it does not play a decisive role.
2. Over the last 30 years, the ILU degree in each city has shown an overall increasing trend, with the highest growth rate occurring between 2000 and 2010. Among them, Beijing had the highest degree in 2018, followed by Tianjin, Zhengzhou, Jinan, and Shijiazhuang. Moreover, by analyzing the cities’ growth in economic, social, and ecological aspects of ILU, it can be concluded that cities with a greater increase in economic aspect are more likely to achieve a higher level of ILU.
3. In terms of the time dimension, the dominant factor affecting the ILU of all cities changed with the city’s development. It shifted from the output-value proportion of secondary and tertiary industries in the early stage to the economic density in the late stage. In terms of the space dimension, the dominant factor varied from cities. In Beijing and Tianjin was economic density, and in Jinan and Zhengzhou was development intensity, and that of Shijiazhuang was population density. By referencing high-level ILU cities (e.g., Beijing, Tianjin), it is evident that improving the economic output efficiency of land and the city’s economic strength is the best way to promote the ILU degree at this time.
4. There will still be a difference in ILU levels between these cities. Cities with high ILU degrees at the current, such as Beijing, would have their ILU increase slowly from 2020 to 2035, while Zhengzhou and Jinan, whose ILU degree has been in the midstream recently, their degree will grow the most among the cities during 2020–2035. Although the ILU level will still vary between cities in the future, this variation should be less than at this stage.

As the conclusions show that the built-up land area is not decisive for the ILU, we recommend that the government strictly control the total amount of built-up land in cities to avoid excessive urban sprawl, and the ILU should be increased by improving the land use efficiency. Specifically, different cities should determine the development strategies according to their current ILU level. For the high-level ILU cities (e.g., Beijing, Tianjin), which are stronger in economic aspect but weaker in social and ecological aspects. These cities should maintain their economic strength while improving their social and ecological strengths, such as increasing road density and urban green spaces. For the low-level ILU cities (e.g., Shijiazhuang), their economic, social, and ecological strengths are not outstanding. The Geodetector results show that improving the economic strength is an important way to improve their ILU level rapidly. Therefore, these cities should increase their economic density by utilizing fewer land resources to support higher quality and more efficient economic development. By implementing these measures, these cities will achieve a rapid increase in the ILU in the future, as predicted by the results.

## Data Availability

All data generated or analyzed during this study are included in the article.
